# F147. RESTING STATE NETWORKS ALTERATION IN PANTOTHENATE-KINASE ASSOCIATED NEURODEGENERATION (PKAN)

**DOI:** 10.1093/schbul/sby017.678

**Published:** 2018-04-01

**Authors:** Gianluca Mingoia, Peter Stoeter, Rea Rodriguez-Raecke

**Affiliations:** 1 RWTH Aachen University; 2 CEDIMAT; 3, Uniklinik RWTH Aachen

## Abstract

**Background:**

While functional MRI and PET studies have shown altered task-related brain activity in PKAN, we want to find such differences also in the resting state (RS).

Here we used ICA based analysis to investigate RS fMRI data to compare connectivity of 11 well known networks (Auditory, Cerebellum, Default Mode Network (DMN), Exectutive Control, Fronto-parietal 1, Fronto-parietal 2, Salience, Sensorimotor, Visual1, Visual2, Visual3 network) between patients with PKAN and healthy controls suggesting deficits in related neuropsychological functions.

**Methods:**

We obtained RS fMRI series (3T, 3x3x3mm resolution, 45 slices, TR 2s, 300 volumes) in 17 PKAN patients but 3 were discarded because of excessive movement, (mean age 17.2a±7.1) on stable medication and 15 healthy controls (22.5a±8.3).

Subjects were asked to lie in the scanner keeping eyes closed with no further specific instructions. Data were pre-processed; we applied FSL MELODIC (pICA) yielding IC, we used FIX to auto-classify ICA components which represent artifacts and an automated routine to select for each subject the component matching the anatomical definition of resting state networks.

SPM12 was used for second level analysis, we used two sample t-test to compare networks functional connectivity between groups. In addition, we used multiple regression to correlate RS networks activity components with Dystonia score.

**Results:**

Our method reliably identified all networks in every control and patients. We found significant differences in the anatomical pattern of areas. Patients showed decreased functional connectivity in comparison to healthy controls in portions of Fronto-parietal 1, Fronto-parietal 2 and Visual1 networks; in addition, patients showed increased functional connectivity in comparison to healthy controls in portions of Cerebellum, DMN, Executive Control, Salience and Visual1 networks. Finally, significant correlation was found between dystonia score and functional connectivity of Cerebellum, Fronto-parietal1, Fronto-parietal2, Salience, Sensorimotor and Visual2 networks.

**Discussion:**

Well known resting state networks were reliable identified from RS fMRI in PKAN patients. The differences in anatomical distribution point to possible alterations in functional connectivity in PKAN, which suggests disruption in cerebellum, DMN, fronto-parietal, salience and visual activity. Correlations with dystonia suggest a direct relation to motor items, which would support a clinical significance of altered RS networks activity.

